# Diagnosing and tracing the pathogens of infantile infectious diarrhea by amplicon sequencing

**DOI:** 10.1186/s13099-019-0292-y

**Published:** 2019-04-06

**Authors:** Haiyan Liu, Mingzhang Guo, Yuanchunzi Jiang, Yanhua Cao, Qingzeng Qian, Xiaoyun He, Kunlun Huang, Jianwei Zhang, Wentao Xu

**Affiliations:** 1 School of Public Health, North China University of Science and Technology, Tangshan, Hebei China; 2 Beijing Advanced Innovation Center for Food Nutrition and Human Health, College of Food Science & Nutritional Engineering, China Agricultural University, Beijing, China; 3 Physical Education Department, Tangshan Normal University, Tangshan, Hebei China; 4 Laboratory of Food Safety and Molecular Biology, College of Food Science and Nutritional Engineering, China Agricultural University, No. 17, Qinghua East Road, Haidian District, Beijing, 100083 China

**Keywords:** Infantile infectious diarrhea, Pathogens detection, Amplicon sequencing

## Abstract

**Background:**

Metagenomic methods have been widely applied to study the relationship between gut microbiota and human health. To test whether metagenomic amplicon sequencing could be an effective method to diagnose and trace the pathogens of infantile infectious diarrhea, the fecal samples of 20 diarrheic and 13 healthy infants were collected. After 16S rDNA amplicon sequencing, diversity analyses were carried out. The relationship between the pathogens of the gut microbiota and geography of patients was analyzed.

**Results:**

The diversity of the gut microbiota in diarrheic infants was significantly lower than that of the gut microbiota in healthy ones and that, the composition of gut microbiota in the diarrheic group was significantly different than that of the gut microbiota in the healthy group. The results also indicated that in some of the patients, the amounts of *Escherichia coli* were significantly increased in the diarrheic infants, which was in agreement with the result of the qPCR analysis. Using a geographical map, we found some patterns between pathogen source and geographical location. This is helpful for an early warning of the disease.

**Conclusions:**

The method of using high-throughput DNA sequencing and a comprehensive and deep data analysis can be a new strategy to detect and trace pathogens in infantile infectious diarrhea.

*Trial registration* Diagnosing and tracing the pathogens of infantile infectious diarrhea by amplicon sequencing, ChiCTR-DDD-1701088, Registered 16 March 2017-Retrospectively registered, http://www.chictr.org.cn/showproj.aspx?proj=18477

## Background

Worldwide, infantile infectious diarrheal diseases constitute a cardinal health problem in developing countries where both morbidity and mortality rates are very high [1]. Globally, 7.6 million children under the age of 5 died in 2010 [2]. Infectious diarrhea accounts for 10.5% of all deaths [3]. Infectious diarrhea is defined as ≥ 3 loose stools in the preceding 24-h period [4–7]. Infectious diarrheal disease is the result of infection by any of a number of different bacterial, fungal and parasitic pathogens. Many factors result in the high incidence of infectious diarrhea in young children [8–11]. Infectious diseases are caused by various pathogens. For an effective treatment, the clinical detection and identification of pathogens have become primary tasks. Traditionally, the detection methods include culture, microscopy and biochemical tests [12, 13]. Conventional examination protocols usually require substantial amounts of labor, time, and skill [8, 14, 16], thus forming an obstacle to a prompt diagnosis [15, 16]. Mixed infections are common, but they are difficult to interpret by traditional methods [17].

Currently, high-throughput sequencing analysis of the amplified 16S rDNA gene allows us to detect known and unknown pathogens [18]. Unlike traditional culture methods, this is a comprehensive, non-cultivable, non-targeted, quantitative detection strategy [19]. Some metagenomics’ studies of feces from a single or a few patients have shown the feasibility of detecting bacterial pathogens [20]. However, cohort studies of detecting pathogens by metagenomics are relatively lacking, and strategies for cohort metagenomics data analysis for diagnosis purposes haven’t been fully developed, which may impede the application of 16S rDNA gene sequencing in clinical bacterial diagnosis. Here, we show an example study of the amplicon-sequencing-based detection of pathogens in individuals from an infantile infectious diarrhea cohort. In this study, we analyzed the potential pathogen in the patients by comparing their gut microbial compositions with those of local, healthy infants.

## Methods

### Participants

Diarrheic infant patients, including seven females and thirteen males, were recruited from Tangshan Maternal and Child Health Care Hospital. Inclusion criteria were diarrheal episodes ≥ 3 times per 24 h and an admission to the hospital within 7 days of the onset of symptoms. All the infants enrolled in this study were diagnosed as bacterial enteritis, and viral enteritis samples have been excluded according to the clinic diagnose in the hospital. The healthy control group included seven female and six male infants with a similar age range to that of the patient group, and the control group participants were not related to the diarrheic infants. All parents signed parent consent forms.

### Fecal samples collection, DNA extraction and 16S rDNA sequencing

Fresh fecal samples of diarrheic and healthy infants were collected in sterile fecal collection tubes and frozen at − 80 °C. Microbial genomic DNA was extracted from each fecal sample using a previously described method [21]. The V3–V4 region of 16S rDNA from each sample was amplified by PCR using the bacterial universal primer pair 341F(CCTAYGGGRBGCASCAG)/806R(GGACTACNNGGGTATCTAA T). The library was constructed using a TruSeq^®^ DNA PCR-Free Sample Preparation Kit (Illumina, San Diego, USA) and high throughput sequencing was conducted using a HiSeq 2500 platform according to the manufacturer’s instructions.

### Bioinformatic analysis

Raw data were assigned to samples based on their unique barcode and truncated by cutting off the barcode and primer sequence. Paired-end sequencing reads were merged using FLASH to get the raw tags [22]. Then the raw tags were filtered by QIIME v1.7.0 [23] (http://qiime.sourceforge.net/) to remove low quality tags and by UCHIME Algorithm [24] to remove chimeric sequences. OTUs (operational taxonomic units) were picked from the remaining tags using Uparse v7.0.1001 [25] at a sequence similarity threshold of 97%, and the representative sequence of each OTU was annotated with RDP Classifier v2.2 [26] and Green Gene Database [27]. The OTU abundance data was normalized to 20,570, corresponding to the sample with the least sequences. All subsequent analyses were performed based on this normalized data.

For the diversity analysis, Bray–Curtis similarity coefficients were calculated based on OTUs data and plotted on a nonmetric multidimensional scaling (NMDS) graph to show the similarity among samples using the software PAST version 3.11 [28]. Analysis of similarities (ANOSIM) was performed with the distance measure set as Bray–Curtis. The Simpson index was calculated to indicate the α-diversity of each sample. The Bray–Curtis index was calculated to indicate the β-diversity for the healthy and diarrheic groups.

The OTU abundance data of healthy infants were used to form a healthy gut microbiota dataset of local infants. To find the key OTUs of the gut microbiota from diarrheal infants that could distinguish between the microbiota from the healthy infants and the microbiota from the infected infants, LEfSe analysis was conducted between the healthy gut microbiota dataset and OTU abundance from individual diarrheal patients. Due to the requirement of LEfSe that each class must contain more than one sample, the OTU data of individual patients were duplicated, which may have some minor effects LDA effect size values of OTUs. The Pathogenic Bacteria Database (http://www.globalrph.com/bacterial-strains.htm) was referred to annotating the pathogenic potential of bacteria. The cluster analysis of healthy and diarrheic infants based on the abundance of potential pathogens was conducted using PAST.

### Analysis of the virulence gene of fecal microflora by real-time quantification PCR methodology

The culture conditions of *E. coli* O157 cells that were described by Leo Heijnen were chosen [29]. Briefly, *E. coli* O157 cells were grown in 5 ml m-TSB broth containing 0.02 g l^−1^ novobiocin for 8 h at 37 °C with agitation. The concentration of *E. coli* O157 colony forming units (CFU) in this suspension was determined by plating dilution series of the suspension on m-TSB (Trypticase Soy Broth Modified) agar plates (Beijing Foodsafety Biotechnology Co., China) and then counting colonies after 16 h of incubation at 42 °C. The concentration of cells in the suspension was also determined by epifluorescent microscopic counting after staining with acridine orange [30]. The CFU concentration was confirmed to be equivalent to the microscopically determined cell concentration resulting in a thoroughly quantified cell suspension. DNA was isolated and purified with a DNA extraction and purification kit according to the manufacturer’s protocol (Cat. A1120, Promega, USA).

The real-time PCR analysis of samples and *E. coli* O157 was performed with a quantification-PCR core kit (Toyobo) by an ABI Prism SDS 7500 instrument (Applied Biosystem). Standard curves of the 16S rDNA gene and the *rfbE* gene were established as described by Rinttila et al. [31]. The amount of specific bacteria was determined by the interpretation of the threshold cycle values to the standard curve, and the results were expressed as log10 CFU/g feces. The sequences of the primers that were used to detect the 16S rDNA gene of the total bacterial sample, total *E. coli* species and the specific *rfbE* gene of *E. coli* O157 are F(GTAAATATGTGGGAACATTTGG)/ R(GGCCTTTAAAATGTAAACAACGG) [32], F(ACCTGCGTTGCGTAAATA) /R(GGGCGGGAGAAGTTGATG) [33] and F(GTAAATATGTGGGAACATTTGG) /R(GGCCTTTAAAATGTAAACAACGG) [34] respectively. PCR conditions were 5 min at 95 °C, 40 cycles of 30 s at 95 °C, and 1 min at 60 °C. DNA amplification was monitored by measuring the accumulation of fluorescence resulting from the binding of SYBR green to double stranded DNA during the 60 °C incubation step. Finally, melting curve analysis was performed by heating the samples to 95 °C and then cooling them down to 55 °C, followed by stepwise temperature increasing of them using 0.1 °C steps, with a 10 s incubation at every step. The fluorescence was measured continuously throughout this analysis.

### Statistics

The data in this study were analyzed with one-way analysis of variance (ANOVA) using SPSS v18.0 (SPSS Inc., Chicago, IL, USA). The differences were considered significant at P < 0.05. The resulting data were presented as the mean ± SD.

## Results

### Gut microbiota of local, healthy infants

**Fig. 1:**
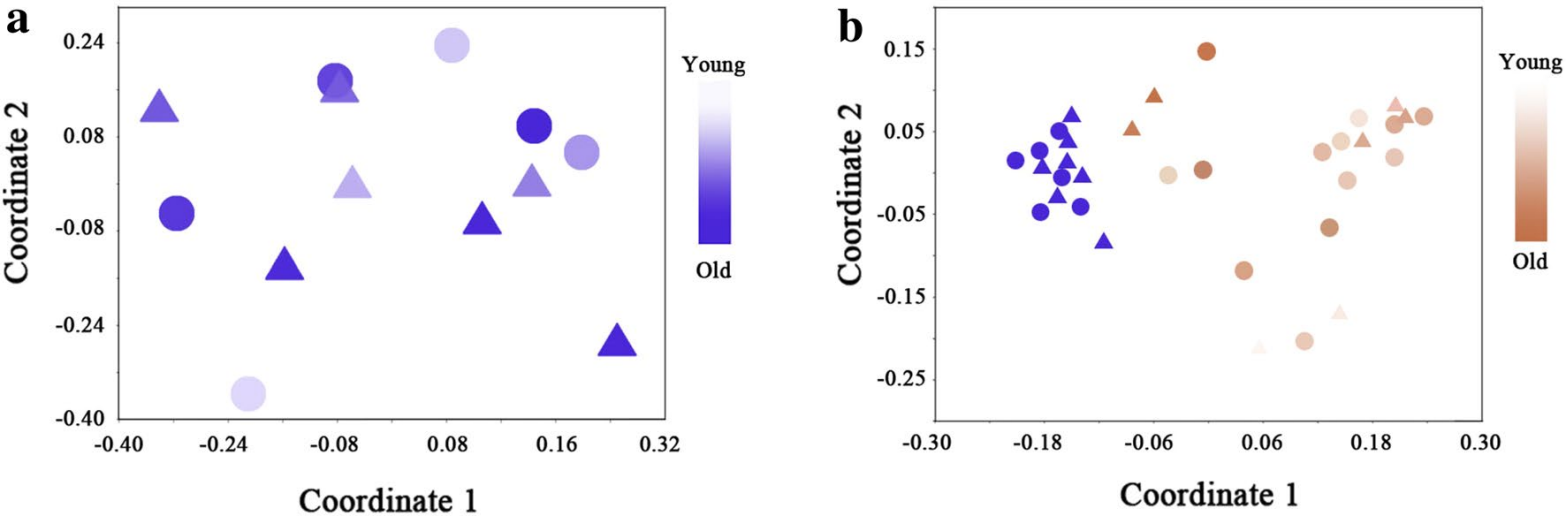
NMDS plot of gut microbiota of healthy and diarrheic infants. **a** The gut microbiota of healthy infants showed no cluster by the factor of gender or age. **b** The gut microbiota of diarrheic infants showed disassociation with those of healthy infants. Blue circular: male healthy, blue triangle: female healthy, brown circular: male diarrheic; brown triangle: female diarrheic

The gut microbiota of humans differs across different ages and geographies [9]. To determine the potential pathogens that cause diarrhea in infants, the gut microbiota of local, healthy infants was first analyzed to get a healthy control profile. Gut microbiota data from seven female and six male infants ranging from 17- to 42-months-old were included in the healthy control profile of this study. The nonmetric multidimensional scaling (NMDS) plot of this healthy gut microbiota showed that there was not a significant cluster between different genders and that no trend with age was observed from the view of a comprehensive community structure (Fig. 1a). Firmicutes, Actinobacteria and Bacteroidetes were identified as the most abundant phyla in the gut microbiota of healthy infants, among which Firmicutes (66.5%) was predominant, followed by Actinobacteria (22.7%) and Bacteroidetes (7.6%). The most abundant OTU included OTU3 (*Bifidobacterium*, 12.8%), OTU6 (*Roseburia*, 9.4%), OTU7 (*Faecalibacterium prausnitzii*, 9.1%), OTU9 (*Ruminococcus bromii*, 5.8%), and OTU2 (*Bifidobacterium*, 4.2%). No known diarrhea-causing bacteria were found to be more abundant than 0.1% in the gut microbiota of healthy infants, and the abundance of opportunistic pathogens in healthy infant gut, such as *E. coli*, *Enterococcus* spp., *Bacteroides fragilis*, *Acinetobacter* was not more than 10% of the total bacteria.

### Community structure and diversity analyses of gut microbiota in diarrheic infants

**Fig. 2:**
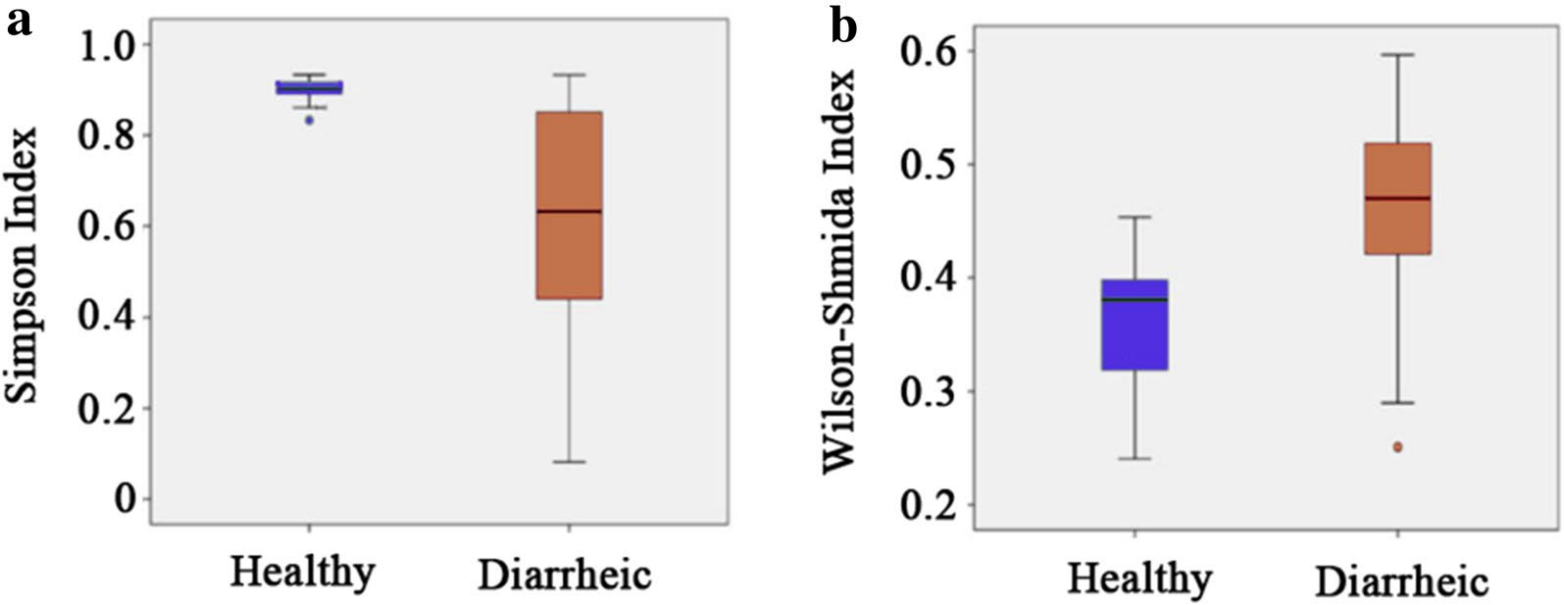
The box plots of **a** α-diversity (Simpson index) and **b** β-diversity (Bray–Curtis index) of gut microbiota of healthy and diarrheic infants. The circles represent the outliers and the asterisks represent the extremes

Fecal samples from twenty diarrheic infants were collected from a local children’s hospital. All of these diarrheic infants were no older than 42 months, and seven of them were females. The gut microbiota of diarrheic infants showed a disassociation with that of healthy infants in the NMDS plot (Fig. 1b), and the ANOSIM analysis implied a significant difference between the gut microbiota of the diarrheic infants and the gut microbiota of the healthy infants (*P* < 0.05). From the NMDS plot of the diarrheic gut microbiota, there was no relationship observed between community structure and age or gender of the infants (Fig. 1a). The distance between the diarrheic infant points in the plot were, on average, much larger than the distance between the healthy infant points (Fig. 1b) and this was confirmed by the result that the β-diversity of the microbiota from the diarrheic group was significantly larger than that of the microbiota from the healthy group (*P* < 0.05, Fig. 2a). However, the α-diversity of the gut microbiota from the diarrheic group was significantly lower than that of the gut microbiota from the healthy group (*P* < 0.05, Fig. 2b).

### Pathogen determination of individual diarrheic infants

**Fig. 3:**
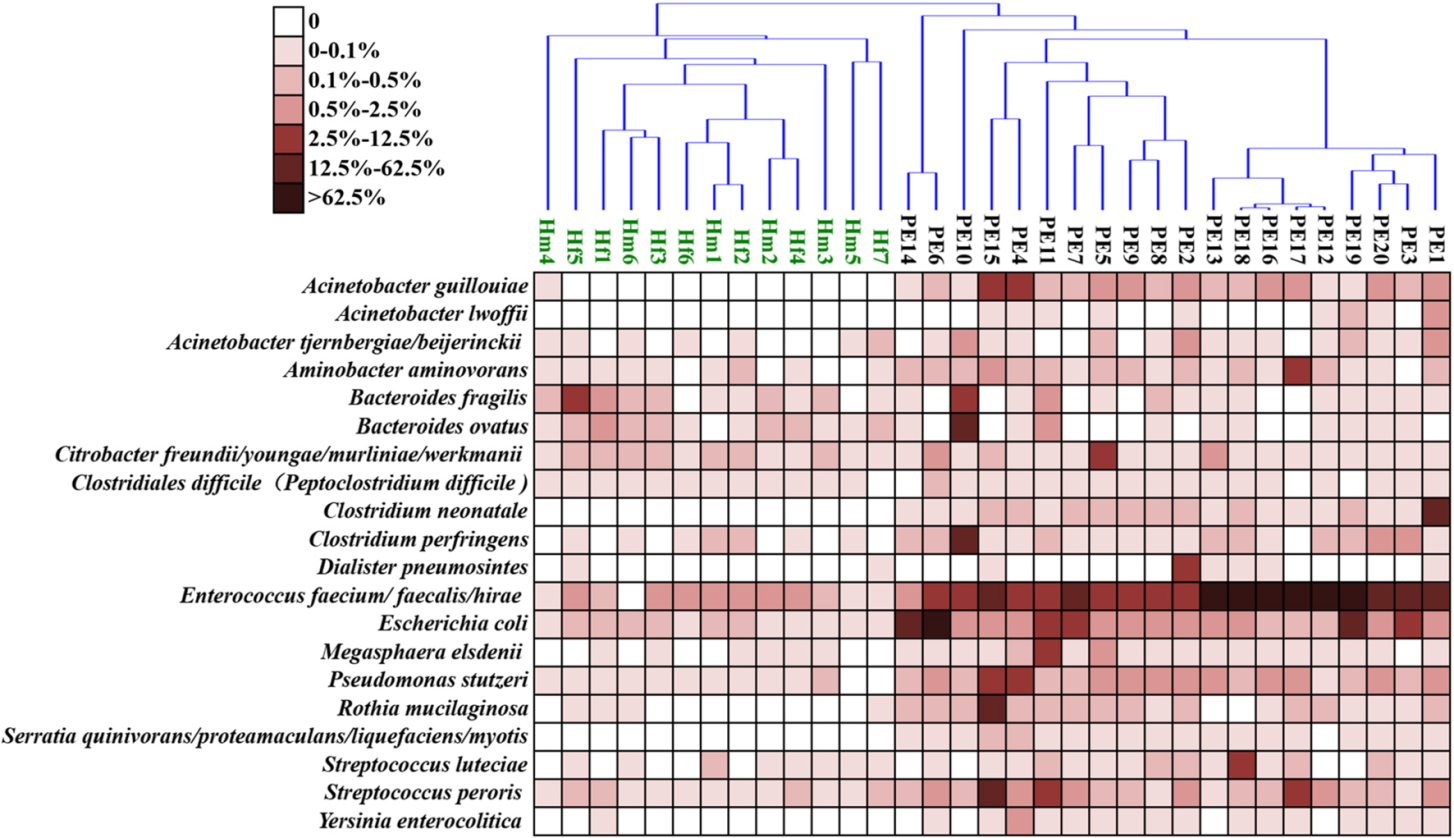
Heatmap of significantly different bacterial genera among healthy and diarrheic infants

According to the obvious increased β-diversity of gut microbiota in diarrheic infants, the diarrhea in the infants in our study was most likely to have a heterogenous etiology. Thus, instead of analyzing the common pathogens in the diarrheic infant group, the potential pathogens were determined by analyzing each individual diarrheic infant. We designed the following two-step procedures to pick up the potential pathogens from individual diarrheic infants. First, the OTU were filtered by LEfSe analysis, and only the OTUs that were more present in at least one of the diarrheic individuals than in the healthy controls and that had value of LDA > 3 were picked out. This step was actually used to find biomarker OTUs in each diarrheic individual. Second, from the filtered OTUs of the first step, two kinds of OTUs were focused on: the OTUs that can be annotated to a known pathogen or opportunistic pathogen and the OTUs that were less than 0.5% abundant in each healthy individual and more than 2.5% abundant in at least one of the diarrheic individuals. The results of this analysis were shown in a heatmap (Fig. 3).

Our results showed that the diarrhea of PE1 was caused by the known pathogen *Clostridium neonatale* [35] which was not found in any of the healthy individuals and was found at an abundance of less than 0.5% in any of the other diarrheic individuals but was presented in PE1 at an abundance of 21.8%. The gut microbiota of PE10 was infected by another clostridium pathogen, *Clostridium perfringens* [36], with an abundance of 16.0%, followed by two Bacteroides species, *Bacteroides ovatus* (14.4%) and *Bacteroides fragilis* (10.6%). PE4 and PE15 showed similar pathogen combinations including *Acinetobacter guillouiae* (5.4% and 5.6%), *Pseudomonas stutzeri* (12.2% and 10.3%), and *Enterococcus* spp., with the former two bacteria in distinctively high abundances compared with the gut microbiota of the healthy infants and other diarrheic infants. PE15 presented with higher abundances of *Rothia mucilaginosa* (15.4%) and *Streptococcus peroris* (15.8%), while PE4 did not. *Yersinia enterocolitica* was detected in PE4 (0.54%), while in another subject, this percentage was less than 0.1%. *Escherichia coli* dominated the gut microbiota of PE6 (76.0%) and PE14 (57.1%), and except for PE12, PE16 and PE 17, *E. coli* showed significant difference in all of the diarrheic infants. *Enterococcus* spp. dominated the gut microbiota of PE1 (55.0%), PE3 (53.1%), PE12 (95.2%), PE13 (73.2%), PE16 (91.2%), PE17 (96.3%), PE18 (92.0%), PE19 (67.3%), and PE20 (57.0%). *Dialister pneumosintes* (2.8%) in the gut microbiota of PE2, *Citrobacter* spp. (3.6%) in the gut microbiota of PE5, *Megasphaera elsdenii* (8.1%) and *S. peroris* (8.6%) in the gut microbiota of PE11 were significantly higher than those in healthy control group. However, no obvious changes in the abundances of potential bacterial pathogens were found for PE7, PE8, and PE9.

### Relationship of gut microbiota pathogen and geography of patients

**Fig. 4:**
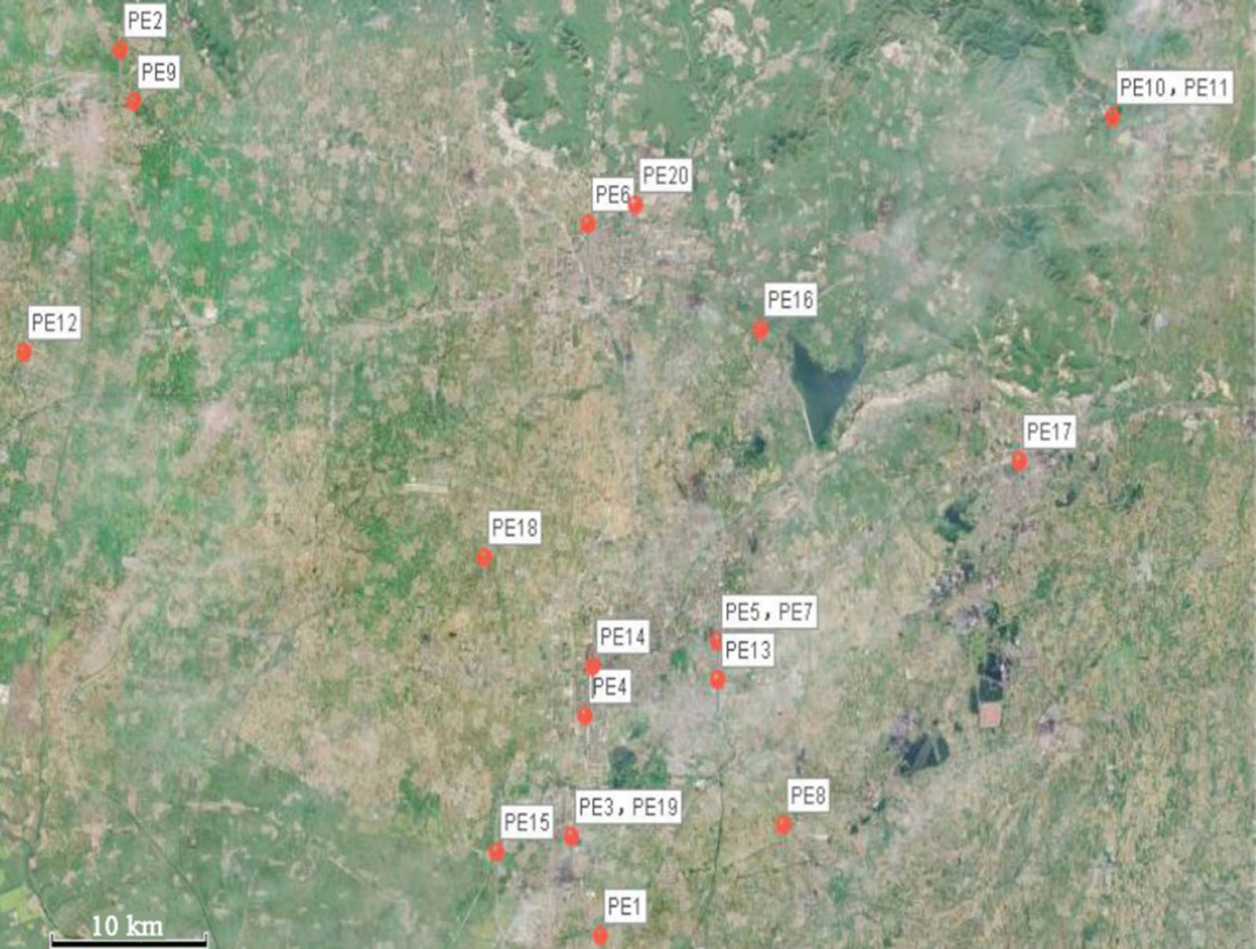
Geography map of patients. Each red point presents the habitation of one patient

From the cluster analysis of healthy and diarrheic infants based on the abundance of potential pathogens, PE3 and PE19 showed clusters with distinctive characteristics in the abundance of *Enterococcus faecium* and *E. coli.* Interestingly, from the geographical map of patients (Fig. 4), the habitations of PE3 and PE19 showed a close geographical distance. Additionally, PE5 and PE7 formed a cluster (Fig. 3), and these two patients live in the same community (Fig. 4). This relationship indicated that there might be some common source of the pathogen around these geographical points.

### qPCR analysis of the virulence genes in the gut microbiota of diarrheic infants

**Fig. 5:**
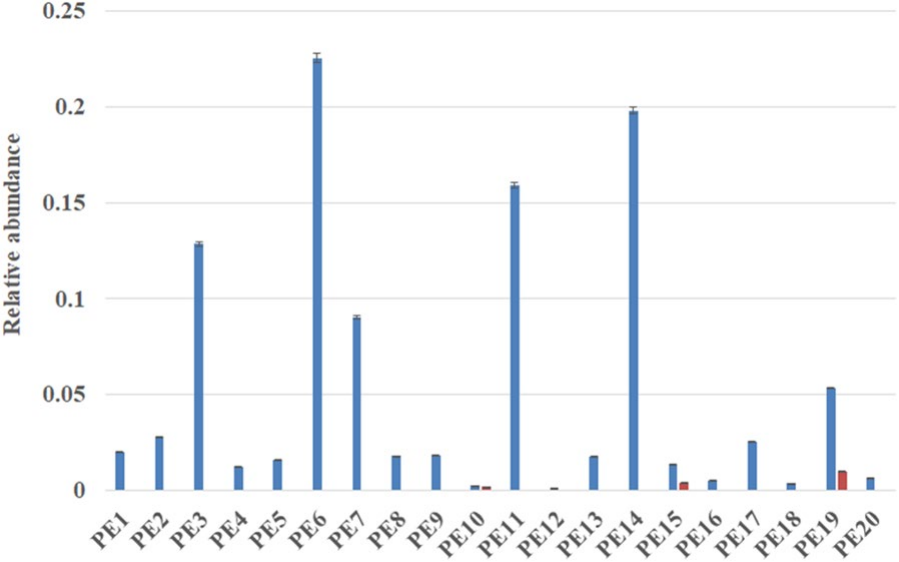
The relative abundance of *E. coli* species (blue bar) and *E. coli* O157 (red bar)

To further analyze the presence of pathogenic *E. coli* in the gut microbiota of patients, the abundance of the virulent gene *rfbE* was detected by qPCR. Standard curves of the 16S rDNA and *rfbE* genes displayed a good linear relationship between Ct and the starting amount of DNA (R^2^ > 0.996), with an amplification efficiency ranging from 97 to 110%. From the results of qPCR, PE3, PE6, PE7, PE 11, PE14 showed high abundance of total *E. coli* species, while the *rfbE* gene can be detected in only PE 10, PE15 and PE19, and *rfbE*, as a specific gene of *E. coli* O157, can reflect the pathogenicity of *E. coli*. The result revealed that the *E. coli* in PE 10, PE15 and PE19 include both O157 and non-O157 serotype (Fig. 5).

## Discussion

16S rDNA sequencing based metagenomics can accurately detect the changes of gut microbiota composition. Based on the results of high-throughput sequencing, we can further tap into the relative information of the gut microbiota. By means of sequencing and in-depth data analysis described in this study and in addition to finding a causative pathogenic microbe, we analyzed the possible relationship between the microbial population’s diversity and living environment. Our main goal was to provide a new strategy for bacterial pathogen diagnosis and etiological analysis.

A variety of factors, such as environment, geographic district, dietary habits and cultural traditions are closely connected with the composition of gut microbiota [9, 31]. The features of gut microbiomes are unique to different locations. For accurate results, when conducting research involved in determining the diversity of gut microbiota, the above aspects should be considered. Therefore, for the controls in our study, we selected healthy infants from the same district as the infants with infectious diarrhea so that we could ensure a more meaningful result. This study revealed that Firmicutes accounted for the largest proportion of bacteria, which is similar to the findings of previous studies [37].

A pathogen infection would change the microbial community’s configuration. NMDS analysis of Bray–Curtis similarity coefficients can clearly show these changes in microbial community structure [38]. Using ANOSIM analysis, the result of our study revealed the significant difference in microbial community composition between healthy infants and infectious diarrhea infants. The obvious distinction was the result of enteropathogen invasion into the infectious diarrheic infants [11, 17]. In the diarrheic infant group, the points of the NMDS plot presented a more dispersed distribution. Therefore, we can predict that the increased diversity is due to a different host being infected by different enteropathogens.

The above analytical methods have been presented to discover changes in intestinal microbial profile. However, for the diagnosis of the disease, it is essential to find pathogenic bacteria and explore the etiology of symptoms. Thus, we used then LEfSe method. LEfSe is a computational approach to detail biomarker class comparisons, that helps to understand microbial communities and guide biologists or doctors in detecting novel metagenomic biomarkers [17]. The method can support high-dimensional class comparisons with a particular focus on metagenomic analysis [39]. LEfSe, as a strict tool, was utilized to identify dominant OTUs. From the result of the heatmap, the main pathogenic microbe of every sample was clear, which is something that is usually difficult to achieve by conventional examination protocols.

The geographic information system (GIS) can transfer spatial data into the geographic coordinate system or projected coordinate system, and ultimately lead to related data visualization [40]. In disease surveillance, through the analysis of time and the spatial distribution of the disease, we can find the disease propagation law. GIS has been widely applied in many infectious diseases [40, 41]. We labeled the home addresses of the participants on the geographic map. According to the spatial distribution of children, we tried to find the epidemic trend of the disease of infectious diarrhea and provide an early warning of the disease. Therefore, the strategy demonstrates its unique and significant superiority with which other examination procedures cannot compared.

The method of high-throughput sequencing has the characteristics of a comprehensive assessment. It ensures that we can select out all pathogens without leaving any out. For the precise quantitative analysis of some pathogens, the qPCR analysis has unique advantages. In this study, we verified pathogens by further qPCR analysis.

## Conclusions

High throughput amplicon sequencing of fecal samples could be used to determine the pathogen of individual diarrheic infants, and the microbiota pathogen pattern from sequencing data could be combined with geography map to help to realize the process of diarrhea outbreak. These results indicated that high-throughput DNA sequencing combined with comprehensive and deep data analysis can used as a new strategy to detect and trace pathogens in infantile infectious diarrhea.

## Abbreviations

NMDS: nonmetric multidimensional scaling
qPCR: quantitative polymerase chain reaction
OTU: operational taxonomic unit
ANOSIM: analysis of similarities
Ct: cycle threshold
GIS: geographic information system
CFU: colony forming units
ANOVA: analysis of variance
